# Psychometric evaluation of the Patient Health Engagement Scale (PHE-s®) in adolescents with chronic diseases: evidence from Türkiye

**DOI:** 10.3389/fpsyg.2026.1810006

**Published:** 2026-05-12

**Authors:** Dilara Usta, Nebahat Bora Güneş, Guendalina Graffigna

**Affiliations:** 1 EngageMinds HUB – Consumer, Food & Health Engagement Research Center, Catholic University of Sacred Heart, Cremona, Italy; 2 Faculty of Agriculture, Food and Environmental Sciences, Catholic University of Sacred Heart, Cremona, Italy; 3 Department of Pediatric Nursing, Faculty of Nursing, Hacettepe University, Ankara, Türkiye

**Keywords:** adolescents, chronic illness, health psychology, patient engagement, psychological adjustment, self-management

## Abstract

**Aim:**

Patient engagement is increasingly recognized as a key determinant of adolescents’ adaptation to chronic illness; however, developmentally sensitive measures capturing engagement trajectories in youth remain scarce. Although the Patient Health Engagement Scale (PHE-s®) has demonstrated robust psychometric properties in Turkish adult populations, its applicability to adolescents has not yet been examined. The objective of this study was to examine its psychometric properties among adolescents with chronic diseases in a Turkish inpatient sample.

**Methods:**

A cross-sectional psychometric validation study was conducted with 200 adolescents aged 12–19 years hospitalized for chronic conditions in a tertiary pediatric hospital in Türkiye. The PHE-s® was evaluated using a multi-method approach integrating Rasch Partial Credit Modeling, categorical principal component analysis, and confirmatory factor analysis. Internal consistency, test–retest reliability, and differential item functioning across demographic and clinical subgroups were examined. Concurrent validity was assessed through associations with the Child Attitude Toward Illness Scale (CATIS).

**Results:**

Analyses supported a unidimensional structure of the adolescent PHE-s®, with strong item loadings and acceptable model fit across analytic approaches. The scale demonstrated high internal consistency (ordinal *α* = 0.90) and excellent one-week test–retest reliability (ICC = 0.98). Measurement invariance was largely supported across sex, education level, and follow-up attendance, with limited differential item functioning observed by age and medical-device dependence. Patient engagement was moderately and negatively associated with illness attitudes (*ρ* = −0.53, *p* < 0.001), indicating that higher engagement corresponds to more positive illness appraisals while reflecting a related but distinct construct.

**Conclusion:**

The Turkish PHE-s® is a reliable and valid instrument for assessing psychological engagement trajectories among adolescents with chronic diseases in inpatient settings.

## 1 Introduction

Adolescence is a developmental period characterized by rapid psychological and social change, including future orientation, cognitive maturation, and heightened sensitivity to peer evaluation (Chaku and Davis-Kean, 2024; Silvers, 2022). When chronic illness is present, these normative developmental tasks intersect with sustained self-management demands (e.g., symptom monitoring, medication routines, diet, healthcare attendance) and with evolving autonomy negotiations within parent–adolescent relationships (Best and Ban, 2021). Consequently, adolescent self-management is not merely a behavioral issue but a developmental transition embedded in shifting responsibility and control dynamics that can either support or undermine emerging self-regulatory capacities (Branje et al., 2021; Mwangi and van Wyk, 2021).

From a health psychology perspective, this intersection creates a predictable “pressure point”: adolescents are expected to demonstrate competent self-management, even as emotional regulation, executive functioning, and identity processes are still consolidating. Illness-related stressors, such as uncertainty, unpredictability of symptoms, and concerns about normality, may amplify vulnerability and shape adherence patterns and longer-term trajectories (Berkelbach van der Sprenkel et al., 2022; Mwangi and van Wyk, 2021). Barriers commonly include illness-related anxiety, inconsistent routines, motivational fluctuations, and perceived constraints on autonomy, particularly when parental protection intensifies (Dave et al., 2024; Shah et al., 2023). These challenges often peak during the transition from pediatric to adult services, which requires psychological readiness and proactive engagement with care alongside practical skills (Betz and Coyne, 2020; Killackey et al., 2023).

Within this developmental and relational context, patient engagement has been proposed as a core psychological determinant of how individuals adapt to illness and participate in care. The Patient Health Engagement (PHE) model conceptualizes engagement as a dynamic, staged process integrating cognitive, emotional, and behavioral components, describing how people make sense of illness, regulate emotional responses, and increasingly enact proactive health-related behaviors (Graffigna and Barello, 2018). The Patient Health Engagement Scale (PHE-s®) operationalizes this trajectory through four ordinal phases: Blackout, Arousal, Adhesion, and Eudaimonic Project, each describing increasingly advanced modes of psychological adjustment to illness and autonomy in health management (Graffigna et al., 2015). Briefly, Blackout represents a state of emotional overwhelm, where the patient feels unable to process the illness experience; Arousal reflects emerging awareness and initial emotional mobilization; Adhesion describes a phase of engagement with self-management and healthcare routines; and Eudaimonic Project characterizes a fully integrated orientation toward health, in which illness management becomes a part of a meaningful life project (Graffigna and Barello, 2018).

While the PHE-s® has shown robust psychometric properties in adult samples across multiple languages (Graffigna et al., 2015; Magallares et al., 2017; Usta et al., 2019; Zhang et al., 2017), its application to the unique developmental neurobiology and social scaffolding of adolescence remains unexplored. This represents a significant gap, as the psychological drivers of engagement in youth differ substantially from those in adults due to the ongoing maturation of executive function and shifting dependency on family systems. Across these adult validations, conducted in Italian (Graffigna et al., 2015), Chinese (Zhang et al., 2017), Spanish (Magallares et al., 2017) and Turkish (Usta et al., 2019) populations, the PHE-s® consistently demonstrated an unidimensional factor structure with high factor loadings, and good-to-excellent internal consistency (ordinal *α* ranging from 0.80 to 0.89). To the best of our knowledge, no study to date has examined the psychometric properties of the PHE-s® in an adolescent population, leaving a critical gap in the literature regarding its developmental applicability.

However, assessment of adolescent chronic illness is currently dominated by measures that emphasize behavioral preparedness (e.g., activation), transition readiness, or service engagement (Bomba et al., 2018; Killackey et al., 2023; Sebastian et al., 2014). While valuable, these tools primarily index skills and observable behaviors, often underrepresenting emotional adjustment and illness-meaning making. In contrast, the PHE model enables identification of ‘hidden vulnerability’ profiles, a phenomenon where adolescents may exhibit high behavioral compliance (pseudo-engagement) while remaining emotionally overwhelmed or cognitively unintegrated (Graffigna and Barello, 2018). Accordingly, addressing this psychological gap is essential for preventing long-term disengagement during the transition to adult care.

Cultural context further motivates adolescent adaptation work, as autonomy and responsibility of health are negotiated within culturally shaped family systems. In Türkiye, family relationships are characterized by strong interdependence and enduring norms of parental responsibility, which may influence how adolescents experience autonomy in illness-related decisions (Dost-Gözkan, 2021). This cultural familism creates a unique psychological environment where adolescent autonomy is co-constructed rather than individually asserted. While such dynamics can provide emotional security, they may also constrain opportunities for independent self-management. International evidence suggests that parental overinvolvement can impede adolescents’ readiness for autonomous care (Badour et al., 2023), and developmental research in Türkiye reflects similar tensions within broader autonomy-related processes (Alsancak-Akbulut and Kömürcü-Akik, 2024). Validating the PHE-s® in this specific context is not merely a linguistic exercise; it is a necessary step in understanding how cultural interdependence influences the internal psychological transition from ‘being cared for’ to ‘taking charge’ of one’s health. Currently, evidence on adolescents’ engagement processes in Türkiye remains limited, and the lack of a psychometrically evaluated PHE-s® for Turkish adolescents constrains the development of culturally responsive psychological assessments and interventions.

Based on these premises, the aim of the present study was to adapt the PHE-s® for adolescents with chronic diseases and to evaluate its psychometric properties, including content validity, internal consistency, construct validity, and measurement adequacy in a Turkish inpatient adolescent sample. To support validity evidence, associations with attitudes toward illness were examined, given the theoretical relevance of illness appraisal and emotional integration to engagement trajectories. It was hypothesized that while engagement and illness attitudes are theoretically related, the PHE-s® would demonstrate distinct construct validity by capturing the staged, proactive nature of psychological readiness for self-care.

## 2 Methodology

### 2.1 Research design and setting

A cross-sectional psychometric validation study with a short-term retest component was conducted, with reporting guided by the Strengthening the Reporting of Observational Studies in Epidemiology (STROBE) guidelines (Vandenbrouckel et al., 2007) for the observational and sample characterization components, and by the COnsensus-based Standards for the selection of health Measurement INstruments (COSMIN; Gagnier et al., 2025) for the psychometric evaluation components. The study was conducted in inpatient wards of a tertiary pediatric hospital in Ankara, Türkiye. A multi-step procedure that incorporated expert content review and quantitative psychometric testing was employed to adapt the PHE-s® for adolescents and to evaluate its measurement properties.

### 2.2 Sampling and participants

The study population comprised adolescents aged 12–19 years who were hospitalized for chronic diseases between May 2023 and February 2024. A non-probability purposive sampling method was used to recruit volunteer participants from diverse diagnostic categories to ensure broad representation of chronic illness experiences. Inclusion criteria were: (i) being under follow-up for at least 1 year due to a chronic illness, (ii) absence of diagnosed psychiatric disorders or psychiatric medication use, (iii) ability to read, write, and speak Turkish, and (iv) willingness to participate with written parental consent. Exclusion criteria included learning disabilities, insufficient Turkish language skills, withdrawal at any study phase, or failure to meet inclusion criteria at retest. Neurodevelopmental conditions, such as ADHD, were effectively captured under the exclusion criteria for diagnosed psychiatric disorders and psychiatric medication use.

Methodological work emphasizes that adequate sample size in factor analysis depends on design characteristics rather than a fixed minimum N (Ondé and Alvarado, 2020). Given the five-item structure of the PHE-s® and the planned Rasch modeling and Confirmatory Factor Analysis (CFA), we targeted N = 200 to support stable parameter estimation and evaluation of dimensionality. Guidance on Rasch calibration stability indicates that samples of ~200 can provide usefully stable item estimates under typical conditions (Tennant and Küçükdeveci, 2023). During recruitment, 204 adolescents were approached; one declined consent, and three did not meet the inclusion criteria, yielding a final sample of 200 participants.

### 2.3 Research procedure

Eligible adolescents were approached individually, informed about the study, and invited to participate. Recruitment was conducted by the study researchers, independent of clinical staff, to minimize potential coercion arising from the therapeutic relationship. Written informed consent was obtained from adolescents and their parents before data collection. The PHE-s® was re-administered to participants who had consented to attend the test–retest phase 1 week later. A total of 107 participants completed the retest administration. The initial in-person administration took approximately 15 min, and follow-up completion took approximately 5 min per individual.

### 2.4 Instruments

#### 2.4.1 Sociodemographic and clinical information form

Data included age, sex, education level, duration of diagnosis, medication use, medical device dependence, and routine attendance at medical follow-up visits.

#### 2.4.2 Patient Health Engagement Scale

The PHE-s® consists of five items with seven ordered response categories reflecting progressive engagement positions. The 7-point response format was deliberately designed to reduce social desirability bias: as declaring a low engagement position (e.g., Blackout) may be socially undesirable, intermediate response options allow respondents to self-position more accurately between stages (Graffigna et al., 2015). For this reason, to align with the foundational PHE model (Graffigna et al., 2015), the responses were recoded into four distinct ordinal phases: Blackout (1; responses 1–2), Arousal (2; responses 3–4), Adhesion (3; responses 5–6), and Eudaimonic Project (4; response 7), grouping intermediate positions into the preceding engagement stage and yielding clinically interpretable profiles of an adolescent’s emotional, cognitive, and behavioral integration of their illness. Given the ordinal nature of the scale, the final PHE-s® score is expressed as the median of the five recoded item responses, which provides a more appropriate summary measure than the mean for ordinally structured data (Graffigna et al., 2015). Example items include “When I think about my illness, I feel overwhelmed by emotions” and “I find my life meaningful despite my illness.” The original scale demonstrated strong reliability (ordinal *α* = 0.85), and the Turkish adult version showed good internal consistency (ordinal α = 0.80) (Graffigna et al., 2015; Usta et al., 2019). It is important to note that the psychometric analyses, including CFA, operate on the five items as continuous indicators of a single latent construct; the engagement phase categories serve clinical profiling purposes rather than representing separate subscales.

#### 2.4.3 Child Attitude Towards Illness Scale

The Child Attitude Toward Illness Scale (CATIS) assesses children’s attitudes toward chronic illness using 13 semantic differential items (Austin and Huberty, 1993). The scale is rated on a 5-point semantic differential scale anchored by opposing adjectives (e.g., “very good”–“very bad,” “very sad”–“very happy”). Items can be tailored to the child’s specific illness (e.g., “How good or bad do you feel it is that you have [specific illness]?”). Scores are calculated by summing responses and dividing by 13, with higher scores indicating more negative attitudes toward the illness (Austin and Huberty, 1993). The original CATIS demonstrated good internal consistency, with Cronbach’s alpha values ranging from 0.74 to 0.86 (Austin and Huberty, 1993), and the Turkish adaptation showed acceptable reliability (Cronbach’s *α* = 0.79) (Ersun and Bolışık, 2012). In this study, the CATIS showed strong internal reliability, with Cronbach’s alpha of 0.91.

### 2.5 Content validity of the PHE-s® among the adolescent population

The linguistic and cultural adaptation of the PHE-s® for Turkish adult patients with chronic diseases had been previously completed (Usta et al., 2019). To ensure the applicability of the PHE-s® for adolescents, a content validity assessment was conducted to evaluate the relevance, clarity, and cultural appropriateness of the items in this age group. An expert panel of seven specialists in pediatric chronic care examined the items of the Turkish-adapted PHE-s®, assessing conceptual and content equivalence, grammatical and spelling accuracy, and rating item readability, clarity, comprehensibility, and relevance using a four-point ordinal scale (1 = not relevant; 4 = very relevant and concise). Following the expert review, the revised scale was administered to 10 adolescents with chronic diseases to assess item clarity and comprehensibility. These adolescents were excluded from the final validation sample to minimize potential bias in the psychometric evaluation.

### 2.6 Statistical analysis

All analyses were conducted using IBM SPSS Statistics version 29 (IBM Corp., Armonk, NY, USA), R version 4.4.3, and RStudio. Descriptive statistics were used to characterize the sample; categorical variables were summarized as frequencies and percentages, and continuous variables as means and standard deviations. Content validity indices were calculated at the item level (I-CVI) and scale level (S-CVI/Ave) to quantify the extent to which items adequately represented the construct (Polit-O’Hara and Yang, 2016). An I-CVI ≥ 0.80 was considered acceptable. Given the ordinal structure of the PHE-s®, median values and the Shannon Entropy Index were calculated to evaluate the distribution and informational richness of item responses. No missing data were observed; therefore, all analyses were conducted on complete cases.

A multi-method analytic strategy was adopted to triangulate measurement properties across Item Response Theory and Classical Test Theory frameworks. First, the Partial Rasch Credit Model (PCM) was applied to assess unidimensionality and item functioning (Masters, 2016). The Person Separation Index (PSI) was used to evaluate the reliability of person estimates under the PCM. Infit and Outfit mean square (MNSQ) statistics, item difficulty estimates (logits), and Chi-square statistics were used to evaluate item fit, with values near 1.0 indicating adequate fit to the latent construct. Crucially, category threshold ordering was inspected to verify that the four-phase ordinal scoring produced ordered thresholds and distinct categories.

To evaluate the stability of the measure across demographic subgroups, an essential step for adolescent psychological assessment, Differential Item Functioning (DIF) was examined using Ordinal Logistic Regression models to ensure measurement invariance across sex (male vs. female), age (12–14 vs. 15–19 years), education level (elementary vs. high school), medical device dependence (user vs. non-user), and regular medical follow-up attendance. For each item, the group variable was entered as a covariate while matching for the mean PHE-s® score.

For exploratory dimensionality analysis, Categorical Principal Component Analysis (CATPCA) with optimal scaling was performed, specifying a one-factor solution consistent with the theoretical unidimensionality of the PHE model. Variable Principal Normalization was used to optimize item–component associations, and no rotation was applied because a single factor was tested. Eigenvalues and the proportion of explained variance were examined, with eigenvalues greater than 1.0 providing empirical support for factor retention.

To further evaluate the latent structure, CFA was conducted using a robust weighted least squares mean and variance (WLSMV) estimator, which is recommended for ordinal indicators with limited response categories. Model fit was assessed using multiple indices: Comparative Fit Index (*CFI* ≥ 0.90), Tucker–Lewis Index (*TLI* ≥ 0.90), Root Mean Square Error of Approximation (*RMSEA* ≤ 0.08), and Standardized Root Mean Square Residual (*SRMR* ≤ 0.08). Factor loadings ≥ 0.40 were considered indicative of adequate item contribution to the latent construct. Modification indices were inspected to identify potential sources of misfit; however, model modifications were only accepted when theoretically justifiable and did not compromise the conceptual integrity of the scale. Internal consistency of the PHE-s® was evaluated using the ordinal alpha coefficient, which is considered more appropriate than Cronbach’s alpha for ordinal data (Bonanomi et al., 2015). Ordinal alpha values ≥ 0.70 were interpreted as acceptable, and values ≥ 0.80 as indicating good internal consistency. Average Variance Extracted (AVE) was also computed as the mean of the squared standardized CFA factor loadings to assess convergent validity, with values ≥ 0.50 considered acceptable (Hair et al., 2022).

Concurrent validity was examined by analyzing the association between PHE-s® scores and CATIS scores using Spearman’s rank correlation coefficient (*ρ*), given the ordinal nature of the PHE-s® items. Based on theoretical assumptions, we hypothesized that higher engagement would be associated with less negative attitudes toward illness, and therefore expected a negative correlation of at least moderate magnitude (|ρ| ≥ 0.30).

Test–retest reliability was assessed using the Intraclass Correlation Coefficient (ICC) based on a two-way mixed-effects model with absolute agreement, which assumes fixed raters and is appropriate for evaluating the stability of repeated measures over time (Landers, 2023). Intraclass Correlation Coefficient values higher than 0.750 were interpreted as indicating good temporal stability. The interval between test and retest administration was 1 week, chosen to minimize recall bias while assuming clinical stability. All statistical tests were two-tailed, and *p*-values < 0.05 were considered statistically significant.

## 3 Results

### 3.1 Content validity

The I-CVI values ranged between 0.85 and 1.00, and the S-CVI/Ave was 0.92, indicating excellent content validity. Kendall’s *W* test showed statistically significant agreement among expert ratings (Kendall’s *W* = 0.84, *p* < 0.001). Minor cultural and linguistic refinements were suggested to enhance the scale’s appropriateness for Turkish adolescents. After administering the scale to 10 participants, their feedback confirmed that the items were understandable and appropriate, with no further changes required.

### 3.2 Sociodemographic and clinical characteristics

The sample consisted of 200 adolescents with a mean age of 14.1 years (*SD* = 1.76) and an average illness duration of 4.1 years (*SD* = 4.01). Females represented 51.5% (*n* = 103) of the cohort. Educational attainment was split between elementary (53.0%, *n* = 106) and high school (47.0%, *n* = 94). The primary diagnostic categories included respiratory (24.5%, *n* = 49), endocrine (23.0%, *n* = 46), and neurological disorders (18.0%, *n* = 36). Details of the chronic diseases are presented in the Supplementary Material. A high majority reported regular medication use (92.5%, *n* = 185) and consistent medical follow-up attendance (91.0%, *n* = 182), while 37.0% (*n* = 74) were dependent on medical devices.

The distribution across PHE-s® engagement phases was as follows: Blackout 36.1% (*n* = 73), Arousal 15.0% (*n* = 30), Adhesion 26.0% (*n* = 52), and Eudaimonic Project 22.5% (*n* = 45).

### 3.3 Psychometric properties of the PHE-s®

#### 3.3.1 Descriptive item characteristics

Table 1 presents item-level descriptive statistics. All items covered the full response range (1–4). Skewness values ranged from −0.046 to 0.231, indicating symmetric distributions, while kurtosis values (−1.32 to −1.70) reflected platykurtic response patterns. Shannon entropy values (1.82–1.98) confirmed adequate informational richness and response dispersion.

**Table 1:** Item-level descriptive statistics for ranks on the PHE-s®.

**PHE-s® item**	**Phase score range**	**Minimum**	**Maximum**	**Median**	**Skewness**	**Kurtosis**	**Shannon Entropy**
Item 1	1–4	1	4	2	0.012	−1.322	1.98
Item 2	1–4	1	4	3	0.013	−1.442	1.98
Item 3	1–4	1	4	2	0.231	−1.589	1.87
Item 4	1–4	1	4	2	0.145	−1.701	1.82
Item 5	1–4	1	4	2	−0.046	−1.603	1.94

#### 3.3.2 Partial credit Rasch model

Table 2 summarizes PCM results. Item difficulty estimates ranged from −1.053 (Item 5) to 1.391 (Item 1). Standard errors were low (0.317–0.621). All items demonstrated acceptable fit (infit and outfit MNSQ between 0.6 and 1.4), with Item 3 showing mild underfit (*Infit MNSQ* = 1.266). Item-level Chi-square statistics were non-significant (*p* > 0.260). Inspection of category thresholds revealed ordered, distinct step calibrations across all items, supporting the adequacy of the four-phase scoring structure. The Person Separation Index (*PSI* = 0.903) demonstrated excellent reliability in differentiating engagement levels.

**Table 2:** Partial credit Rasch model.

**PHE-s® item**	**Measure (logits)**	**S. E.**	**Infit MNSQ**	**Outfit MNSQ**	**Chi-Square (df)**	***p*-value**
Item 1	1.391	0.621	0.754	0.681	88.524 (129)	0.997
Item 2	−0.680	0.317	0.726	0.761	98.945 (129)	0.977
Item 3	0.705	0.323	1.266	1.066	138.643 (129)	0.265
Item 4	0.557	0.324	0.928	0.908	117.985 (129)	0.747
Item 5	−1.053	0.318	0.671	0.635	82.557 (129)	1.000

S.E.: Standard Error, MNSQ: Mean square.

Measurement invariance was assessed across demographic and clinical subgroups using ordinal regression. The PHE-s® demonstrated full invariance across sexes, education levels, and regular attendance of follow-up appointments across all items (*p* > 0.05). Partial invariance was observed for age groups (12–14 vs. 15–19) and medical device use. Specifically, Item 2 exhibited significant DIF for age (*p* = 0.038), and Item 3 showed significant DIF for medical device use (*p* = 0.040).

#### 3.3.3 Categorical principal component analysis

As shown in Table 3, all items loaded strongly on the first component (0.879–0.935). The first eigenvalue was 4.03, explaining 80.6% of the variance.

**Table 3:** Factor loadings from CATPCA analysis.

**PHE-s® item**	**Factor loadings from CATPCA - One-factor solution**
Item 1	0.935
Item 2	0.902
Item 3	0.879
Item 4	0.883
Item 5	0.888

CATPCA: Categorical Principal Component Analysis.

#### 3.3.4 Confirmatory factor analysis

Table 4 presents the CFA results. Confirmatory factor analysis was conducted using the WLSMV estimator, and the one-factor model showed good goodness-of-fit indices, supporting the hypothesized factor structure. Fit indices met conventional criteria (*χ^2^*(5) = 17.95; *CFI* = 0.997; *TLI* = 0.995; *RMSEA* = 0.064; *SRMR* = 0.033), indicating that the model was coherent with the data. All standardized factor loadings were high (0.91–0.95), confirming that each item contributed strongly to the latent patient engagement construct. AVE was 0.881, substantially exceeding the 0.50 threshold and indicating strong convergent validity (Table 4).

**Table 4:** Confirmatory factor analysis of the PHE-s®: Standardized estimates.

**Indicator**	**Std. estimate**	**S.E.**	***p*-value**
Item 1	0.954	–	–
Item 2	0.943	0.018	<0.001
Item 3	0.910	0.022	<0.001
Item 4	0.936	0.021	<0.001
Item 5	0.950	0.018	<0.001
AVE	0.881		

The loading of Item 1 was fixed to 1.00 to scale the factor; therefore, its SE and *p*-value are not estimated. AVE: Average Variance Extracted.

#### 3.3.5 Internal consistency

Ordinal alpha for the PHE-s® was 0.90 (95% CI), indicating high internal consistency (Table 5). Alpha values remained high when individual items were removed (range: 0.84–0.88). The removal of any item decreased the ordinal alpha, confirming that all items contribute to the scale’s internal consistency.

**Table 5:** Ordinal alpha via empirical copula if item deleted.

**PHE-s® item**	**Ordinal alpha if item deleted**
Item 1	0.87
Item 2	0.86
Item 3	0.84
Item 4	0.88
Item 5	0.86

#### 3.3.6 Concurrent validity and test–retest reliability

Findings indicated a moderate negative correlation between PHE-s® and CATIS (*ρ* = −0.53, *p* < 0.001), suggesting that higher psychological engagement is associated with more positive attitudes toward illness. Test–retest data were available for *n* = 107 participants. The analysis yielded an ICC of 0.984 (95% CI: 0.979–0.988), indicating high temporal stability of the total PHE-s® score across the one-week interval. The single-measures ICC was 0.862, suggesting strong reliability of a single measurement occasion. The ICC was statistically significant [*F*_(106, 954)_ = 63.726, *p* < 0.001], confirming that the observed agreement is unlikely to be due to chance.

## 4 Discussion

The present study provides the first psychometric validation of the PHE-s® among hospitalized adolescents with chronic illness, offering strong evidence for its structural validity, reliability, and concurrent validity in a Turkish inpatient sample. Across Rasch modeling, CATPCA, and CFA, findings converged on a single latent dimension, consistent with the PHE model’s conceptualization of engagement as an integrated psychosocial process encompassing cognitive, emotional, and behavioral adaptation to chronic illness (Graffigna et al., 2015). In a developmental period characterized by ongoing identity formation, emotion regulation, and autonomy negotiation, this structural coherence suggests that engagement may represent a meaningful psychological stance toward responsibility for illness rather than merely observable adherence or skill acquisition (Ernst et al., 2022). Methodologically, convergence across item-response and classical test frameworks strengthens the interpretability of scores as reflecting a coherent engagement continuum.

Structural validity was supported by acceptable Rasch item fit and strong CATPCA and CFA evidence for a single latent dimension. This pattern aligns with broader psychometric work in adolescent health measurement (Pakpour et al., 2024), in which brief instruments assessing integrated psychological constructs often show high factor saturation (Groskurth et al., 2024; Kenny et al., 2015). The observed unidimensionality is also theoretically coherent: in adolescence, emotional appraisal, cognitive illness representations, and behavioral orientation are developmentally intertwined, supporting engagement as an integrated psychological stance rather than separable domains (Ernst et al., 2022; Silvers, 2022). Accordingly, model evaluation emphasized theoretical coherence, factor loadings, and the convergence of multiple fit indices, consistent with recommendations for short-scale validation (Groskurth et al., 2024).

Differential item functioning analyses largely supported measurement equivalence of the adolescent PHE-s® across sex, education level, and regular follow-up attendance, indicating that group comparisons on engagement are generally interpretable as reflecting differences in the latent construct rather than item bias. This finding is important in adolescent assessment, where many psychosocial constructs show subgroup-dependent interpretations due to developmental heterogeneity and contextual scaffolding (Riglin et al., 2024). However, two instances of partial invariance emerged: Item 2 showed age-related DIF, and Item 3 showed DIF by medical-device dependence. Item 2 captures a shift from emotional disorientation to emotional clarity; the observed age-related DIF likely reflects developmental differences in emotional awareness rather than item bias. Younger adolescents tend to experience and label emotional arousal in more global or less differentiated ways, whereas older adolescents show greater reflective capacity and emotional coherence (Silvers, 2022; Chaku and Davis-Kean, 2024). Item 3, which indexes emotional integration of illness, showed DIF by medical-device dependence, plausibly reflecting increased illness salience and treatment intrusiveness among device-dependent adolescents. Greater bodily visibility and caregiver involvement have been shown to heighten emotional activation and complicate illness integration during adolescence, independent of general coping or engagement levels (Berkelbach van der Sprenkel et al., 2022; Breuner et al., 2023).

Moreover, internal consistency was high, aligning closely with values reported in adult validations of the PHE-s® across Italian, Turkish, Chinese, and Spanish samples (ordinal *α* = 0.80–0.89) (Graffigna et al., 2015; Magallares et al., 2017; Usta et al., 2019; Zhang et al., 2017), and comparable to reliability coefficients observed in other brief psychosocial measures adapted for adolescents with chronic illness. Although very high coefficients and factor loadings can signal item redundancy and restricted construct breadth (Haroz et al., 2020), in the present case, these values likely reflect strong saturation of a common engagement construct rather than problematic redundancy. This interpretation is supported by the PHE-s® design as a staged psychological measure, each item was developed to capture a distinct experiential position along the engagement continuum, and is consistent with the high factor loadings reported in adult versions of the PHE-s® across other populations (Graffigna et al., 2015; Magallares et al., 2017; Usta et al., 2019; Zhang et al., 2017). Nonetheless, future work should formally examine test information functions to determine whether measurement precision is achieved at the expense of construct coverage, and whether item reduction could preserve sensitivity across engagement phases (Steigen et al., 2022).

Concurrent validity was supported by the moderate negative association between PHE-s® scores and CATIS illness attitudes. This effect size is consistent with the expectation that the two constructs are theoretically adjacent but not redundant: CATIS primarily captures valenced illness attitudes/affective appraisal (i.e., how good–bad or distressing the illness feels), whereas the PHE-s® is intended to index a process-oriented psychological readiness trajectory. Because both measures were self-reported and collected within a single assessment context, the observed association may also be influenced by common/shared method variance. This is particularly relevant in inpatient settings, where hospitalization can heighten emotional distress, increase illness salience, and constrain perceived control and autonomy, conditions under which affective appraisal and engagement-related cognitions may become more tightly coupled than in outpatient or community contexts (Berkelbach van der Sprenkel et al., 2022; Breuner et al., 2023). Importantly, the moderate correlation observed here suggests that the adolescent PHE-s® captures a related but distinguishable construct, while still warranting cautious inference about trait-like engagement when assessed during periods of elevated clinical and emotional load.

One-week test–retest reliability was excellent, indicating that engagement scores remain stable across brief clinical intervals and supporting the use of the PHE-s® for monitoring within relatively stable care windows. Importantly, such temporal stability should not be interpreted as evidence of developmental invariance. Adolescence is characterized by ongoing reorganization of affective, cognitive, and social systems, with progressive integration of future orientation, identity commitments, and autonomy striving (Silvers, 2022; Chaku and Davis-Kean, 2024). Longitudinal research is therefore needed to determine whether engagement trajectories shift across developmental stages, clinical events (e.g., exacerbations, rehospitalizations), and healthcare transitions. Contemporary transition literature emphasizes that successful transfer to adult services depends not only on procedural competence but also on internalized responsibility, emotional readiness, and perceived ownership of care (Parfeniuk et al., 2020). Mapping how PHE-s® stages evolve across this period may thus provide critical insight into adolescents’ readiness for autonomous disease management and identify those at risk for disengagement despite apparent behavioral competence.

### 4.1 Strengths and implications

Clinically and conceptually, adolescent validation of PHE-s® is valuable, as existing tools mostly emphasize behavioral competence (activation, readiness skills) over emotional adjustment and meaning-making. By explicitly integrating emotional adjustment and illness meaning-making, the PHE-s® addresses dimensions largely absent from activation-based or readiness-focused measures. Literature reviews of transition readiness measures note substantial heterogeneity in conceptual coverage and psychometric quality, and highlight the need for measures that better capture motivational and psychological readiness alongside skills (Hart and Chisolm, 2023; Parfeniuk et al., 2020; Varty and Popejoy, 2020). Used alongside readiness instruments, PHE-s® staging may help identify “hidden vulnerability” profiles: adolescents who appear behaviorally competent (e.g., knowledgeable, adherent under supervision) but remain emotionally overwhelmed or cognitively unintegrated in their understanding of illness (Cserép et al., 2022; Rohan and Verma, 2020). Incorporating psychological engagement profiling into transition clinics, alongside tools such as diagnosis-specific readiness questionnaires, may support more tailored educational, psychological, and family-based interventions that address both skill gaps and engagement barriers (Bray et al., 2022; Ernst et al., 2022).

The present findings also have implications for culturally situated care in the Turkish pediatric context, where family involvement remains central to chronic illness management. Research on Turkish families managing childhood chronic conditions has shown that parental responsibility, family organization, and shared decision-making are crucial determinants of disease management and quality of life (Baysal et al., 2023). Evidence from Turkish samples indicates that parental dynamics, including overprotective tendencies and collaboration patterns, relate to adolescents’ self-efficacy and quality of life in chronic disease contexts (Celik et al., 2024; Özgüven Öztornacı, 2024). Future work should therefore test measurement invariance across sex, age bands, and illness groups, and should examine whether associations between engagement and outcomes differ by family involvement patterns (e.g., autonomy support vs. control). Such analyses would directly address whether the same engagement score reflects the same latent standing across subgroups and sociocultural contexts.

### 4.2 Limitations

Despite its strengths, several limitations should be acknowledged. First, non-probability sampling from a single tertiary hospital and the inpatient setting may limit generalizability to community samples and may inflate correlations with illness attitudes due to acute stress; replication in outpatient and community samples is therefore warranted. A critical next step is cross-setting validation (outpatient clinics and transition services), including known-groups comparisons by care intensity, to determine whether item thresholds and phase classifications remain stable outside inpatient contexts. Although diagnostic heterogeneity supports transdiagnostic applicability, illness-specific factors were not examined, and future work should test whether disease characteristics moderate engagement profiles. The exclusion of adolescents with psychiatric and neurodevelopmental conditions, such as ADHD, likely resulted in a psychologically healthier sample, limiting generalizability to clinically complex populations where engagement trajectories may differ. The near-complete participation rate may partly reflect cultural attitudes toward healthcare research and academic authority in Türkiye, as well as the hospitalized context; this may limit the generalizability of engagement profiles to populations where willingness to participate in research differs. Partial DIF by age and medical-device dependence was observed but not explored qualitatively; cognitive interviewing could clarify developmental and experiential differences in item interpretation. Because psychometric analyses were conducted on the 4-phase recoding, additional work is needed to directly compare measurement performance against the original 7-category response format and to quantify potential information loss from category collapsing. Finally, concurrent validity was assessed only with illness attitudes, and parental engagement was not measured; future studies should examine broader nomological networks, family-level engagement, and measurement invariance.

## 5 Conclusion

The present study examined the psychometric performance of the PHE-s® among adolescents with chronic diseases to evaluate its suitability for capturing psychological engagement trajectories within a Turkish inpatient context. By integrating item response theory and classical test theory approaches, the study provides comprehensive evidence regarding the scale’s structural validity, reliability, and concurrent validity in this developmental population.

The findings indicate that the adolescent PHE-s® demonstrates a robust unidimensional structure, high internal consistency, and excellent short-term temporal stability. The moderate negative association between engagement levels and illness attitudes further supports the theoretical relevance of psychological engagement to adolescents’ affective appraisal of illness and confirms that the PHE-s® captures a related but distinct construct. Moreover, limited and developmentally interpretable differential item functioning by age and medical-device dependence suggests that engagement trajectories are shaped by developmental stage and illness-related salience, rather than reflecting systematic measurement bias. Together, these results support the conceptualization of engagement as an integrated psychosocial stance encompassing emotional integration, cognitive meaning-making, and proactive orientation toward self-management during adolescence. Overall, the application of the scale may support more tailored psychological, educational, and family-centered interventions, particularly during periods of heightened vulnerability and transition in care. More broadly, this work contributes to the growing literature on patient engagement by demonstrating the feasibility and value of measuring engagement as a staged psychological process within adolescent populations and culturally interdependent healthcare contexts.

## Data Availability

The raw data supporting the conclusions of this article will be made available by the authors, without undue reservation.

## Glossary

AVE: Average Variance Extracted
CATIS: Child Attitude Toward Illness Scale
CATPCA: Categorical Principal Component Analysis
CFA: Confirmatory Factor Analysis
CFI: Comparative Fit Index
DIF: Differential Item Functioning
ICC: Intraclass Correlation Coefficient
I-CVI: Item-level Content Validity Index
MNSQ: Mean Square
PCM: Partial Rasch Model
PHE model: Patient Health Engagement model
PHE-s®: Patient Health Engagement Scale
PSI: Person Separation Index
RMSEA: Root Mean Square Error of Approximation
S-CVI/Ave: Scale-level Content Validity Index
SD: Standard deviation
SRMR: Standardized Root Mean Square Residual
STROBE: Strengthening the Reporting of Observational Studies in Epidemiology
TLI: Tucker-Lewis Index
WLSMV: Weighted Least Squares Mean and Variance adjusted
